# Pregnancy and thrombosis risk for women without a history of thrombotic events: a retrospective study of the real risks

**DOI:** 10.1186/s12959-022-00419-6

**Published:** 2022-10-06

**Authors:** Elisavet Grouzi, Abraham Pouliakis, Αnthi Aktypi, Anna Christoforidou, Paraskevi Kotsi, Georgios Αnagnostou, Aikaterini Foifa, Emmanouil Papadakis

**Affiliations:** 1 Consultant of Hematology, Head of Transfusion Service and Clinical Haemostasis, St Savvas” Oncology Hospital, Athens, Greece; 2 2nd Department of Pathology, National and Kapodistrian University of Athens, ATTIKON” University Hospital, Athens, Greece; 3 Hematology, OLYMPION General Clinic, Patras, Greece; 4 Hematologist of Hematology Department, Bioclinic Salonica, Thessaloniki, Greece; 5 Transfusion Medicine, Director Blood Bank and Hematology Laboratory, Medical School University of Thessaly, General University Hospital of Larissa, Larissa, Greece; 6 Transfusion Service and Clinical Haemostasis, Henry Dunant Hospital Center, Athens, Greece; 7 Transfusion Department, Iaso General Maternity and Gynecology Clinic, Athens, Greece; 8 Thrombosis & Hemostasis clinic Ob/Gyn Hematology, Genesis Hospital, Thessaloniki, Greece

**Keywords:** Low molecular weight heparin, Pregnancy, Venous thromboembolism, Pregnancy complications

## Abstract

**Background:**

During pregnancy and puerperium women are at high VTE risk. Current guidelines recommend dynamic VTE-risk assessment during pregnancy. Based on related RCOG-guidelines we constructed a digital VTE-risk assessment tool: PATrisks (www.PATrisks.com). Using this tool, we retrospectively evaluated the thrombotic risk in 742 women from our previous work, women who received thromboprophylaxis based on clinical experience for A) pregnancy complications, B) IVF treatment and C) prothrombotic tendency, in order to investigate whether that practice was justified according to the PATrisks scoring system for VTE prevention.

**Methods:**

Women with pregnancy complications [Group-A: 445], women who had undergone IVF [Group-B:132] and women with a prothrombotic tendency (thrombophilia, family history of VTE, other) [Group-C:165] were assessed using the PATrisks scoring system for thrombotic risk. The women were assigned into one of the following risk categories: low (score ≤ 2), intermediate (score = 3) and high (score ≥ 4). Further analysis per risk factor type (pre-existing or obstetric) and for various combinations of them, was also performed. We evaluated thrombotic risk early in pregnancy, and in the peripartum period.

**Results:**

The mean risk score antepartum was higher for women in Group B (3.3 in comparison with 1.9 and 2.0 in Group A and Group C respectively). Moreover, the risk score increased significantly postpartum for all Groups. The chi-square test also proved that there was a higher percentage of women at high or intermediate risk in group B compared to C before birth (55.3% vs.26.1% respectively, *p* < 0.0001, OR: 3.5, 95% CI: 2.2 – 5.7) and similarly after birth (85.6% vs. 56.4%, OR: 4.6, 95%CI: 2.6–8.2, *p* < 0.0001). In total 12 (1.6%) out of 742 women experienced thrombotic events, whether pre- or post-partum.

**Conclusions:**

LMWHs are widely prescribed during pregnancy for a number of indications, even when a proven scientific basis for such a practice is lacking. However, a considerable percentage of women were already at VTE-risk according to PATrisks and might have derived an additional benefit from LMWH in the form of VTE prevention. The rational use of these drugs should be optimized by establishing and implementing routine risk assessment for all pregnant women and by providing the necessary education to healthcare professionals.

## Background

Pregnancy-associated Venous thromboembolism (PA-VTE), including deep vein thrombosis (DVT) and pulmonary embolism (PE), is one of the most common causes of maternal morbidity and mortality in developed countries [1].

This is largely attributable to pregnancy-related changes in clotting factors that lead to a state of physiological hypercoagulability [2]. Thus, in comparison to non-pregnant women of the same age, pregnant women have an approximately 4–5 times higher risk of VTE [3]. The risk for VTE rises during pregnancy and peaks in the postpartum period [4].

This thrombotic risk which is anyway elevated by pregnancy increases further if additional intrinsic and extrinsic risk factors for VTE are present in the pregnant woman. Intrinsic risk means the increased proneness of pregnant women towards thrombotic events due to their individual characteristics, while extrinsic risk factors are factors which act on the pregnant woman externally and which situationally increase the risk of thrombosis [5]. Independent risk factors such as being 35 or older, null parity, multiple gestations, obesity, smoking and immobility, increase the risk by a factor of 1.5—2 [6, 7]. Certain other predisposing conditions have been associated with a higher risk of PA- VTE. These include inherited or acquired thrombophilia, a previous history of thrombosis, antiphospholipid syndrome, and other co-morbidities. When these are present, the need for prophylactic anticoagulation should be addressed [6].

The pro-coagulant state of pregnancy could also expose a woman to the possibility of gestational vascular complications (GVCs) (pre-eclampsia, placental abruption, fetal growth restriction (FGR), late and recurrent early miscarriage, intrauterine death and stillbirth), especially in the presence of acquired or inherited thrombophilia [8–10].

Antiphospholipid antibodies (APLA), including lupus anticoagulant (LAC), anticardiolipin antibodies (ACL) and b-2 glycoprotein antibodies (B2GP) are autoimmune antibodies directed against phospholipid-binding plasma proteins and they have been associated with both arterial and venous thrombosis as well as pregnancy morbidities [10].

Low-molecular-weight heparins (LMWHs) are effective thrombo-prophylactic agents; they do not cross the placenta, and they do not appear in breast milk [11–14]. However, the use of LMWH prophylaxis during pregnancy to prevent recurrent adverse pregnancy complications is supported by limited and conflicting evidence [15].

In an attempt to improve Assisted Reproduction Techniques (ART) outcomes many studies have investigated the effects of low-dose aspirin or low-molecular-weight heparin (LMWH), because of their antithrombotic and vasodilatory properties. The biological plausibility of antithrombotic prophylaxis may be represented by a beneficial effect in counteracting existing or developing at risk pro-thrombotic conditions [16]. However, the data are controversial.

In Greece LMWHs are used extensively during pregnancy and puerperium for VTE treatment and prophylaxis and for a variety of other indications as well. In this context, we conducted a cohort study [17] in an attempt to elucidate the clinical practice in our country, with the aim of gaining insights regarding the use of LMWHs during pregnancy and puerperium, describing the indications for use, the diagnostic work-up as well as the safety and efficacy of the treatment.

In our previous work we presented data regarding LMWH use in pregnancy for VTE management and the optimization of pregnancy and ART outcomes, with a sample of 818 women receiving LMWH during 2010–2015. There were 4 groups: those with a history of VTE [Group-A: 76], those with pregnancy complications [Group-B: 445], those undergoing IVF [Group-C: 132] and those with a prothrombotic tendency (thrombophilia, family history of VTE, other) [Group-D: 165].

For Group A, the administration of thromboprophylaxis has been well supported by studies and is recommended by existing guidelines. However, for Groups B, C & D clinical decisions about thromboprophylaxis management were based mainly on clinical experience. Specifically, for Group B, the use of LMWH prophylaxis during pregnancy to prevent recurrent GVCs is supported by limited and conflicting evidence and thus it is not recommended at present by guidelines with the exception of women with APLA. For Group C, for the women on IVF, the data are controversial and there is no clear recommendation. While for Group D, the inherited thrombophilia by itself does not necessitate thromboprophylaxis during pregnancy and puerperium.

When dealing with pregnant women, apart from clinical experience, further considerations come into play and all known intrinsic and extrinsic risk factors of the pregnant woman must be taken into account. For this reason, we constructed a digital VTE-risk assessment tool – PATrisks (www.PATrisks.com) based on related RCOG-guidelines [18]. The elevated baseline pregnancy-associated VTE risk necessitates VTE risk assessment in early pregnancy, at delivery, and if the risk factors change.

In order to further analyze our extended available data to gain more insights into the rationale behind LMWHs use in pregnant women with no clear recommendation for thromboprophylaxis, we proceeded in a post-hoc analysis of our previous work cohort and evaluated them for VTE risk retrospectively using PATrisks.

The aim of this study is.

- To investigate if the administration of LMWH in pregnant women of Groups B, C & D (which in this study have been renamed as Groups A, B & C respectively) was justified by the scoring system used for VTE prevention
- To assess the women’s status and to provide details concerning thrombophilia

## Methods

This study adds to the results of our previous study [17], a multicenter, retrospective study that addressed the issue of LMWH use in pregnant women in Greece, and which used as its subjects 818 pregnant women receiving LMWH for prophylaxis based on clinical experience. For the purpose of our post-hoc analysis, women with pregnancy complications [Group-A: 445], those undergoing IVF [Group-B: 132] and those with a prothrombotic tendency (thrombophilia, family history of VTE, other) [Group-C: 165] were assessed via the PATrisks scoring system in order to evaluate their thrombotic risk. Women with a personal history of VTE [categorized as Group-A: 76, in our previous study], were not analyzed since all these women were by definition in a high risk category (scoring 4 in PATrisks) and therefore the administration of thrombophylaxis was clearly recommended anyway.

The risk score was calculated retrospectively and women were assigned into one of the following risk categories: low (score ≤ 2), intermediate (score = 3) and high (score ≥ 4). Further analysis per risk factor type (pre-existing or obstetric) and combinations of them, was also performed; we evaluated the risk first early in pregnancy, and secondly in the peripartum period, as many risk factors are associated with delivery.

Furthermore, we conducted a detailed descriptive analysis of the incidence of thrombophilic defects. The thrombophilic defects were grouped into four categories: a) high risk hereditary thrombophilias including AT III deficiency (< 70%), homozygous FV G1691A, homozygous FII G20210A, combined heterozygous FV G1691A & FII G20210A, Protein C (PC, < 70%) & Protein S (PS, < 60%) deficiency, b) low risk hereditary thrombophilias including heterozygous FV G1691A & heterozygous FII G20210A, c) acquired thrombophilia including APLA and d) other low-risk thrombophilias including MTHFR andPAI4G.

### Statistical analysis

The statistical analysis was performed by the SAS for Windows 9.4 software platform [19] (SAS Institute Inc., NC, U.S.A.). The demographic and clinical data of the patients at the baseline were presented via descriptive statistics. Specifically, as the mean value and standard deviation (SD) and for reasons of completeness the median value and the values for 25% and 75% percentiles were also reported, for the qualitative data, frequencies and percentages were presented. Comparisons between two or more groups for the categorical parameters were performed using the chi-square test [20]. For the quantitative parameters (for example the women’s age) the Mann–Whitney U test was applied for comparison between two groups or the Kruskal–Wallis test when more than two groups were involved [19] respectively; as normality was not always certain. For paired analysis, i.e. to compare the PAT risk pre and post-partum, the Wilkoxon Signed Rank test was applied. The significance level (*p*-value) of the study was set to 0.05 and all tests were two-sided.

## Results

### Study population

In terms of the baseline and treatment characteristics between women with pregnancy complications [Group-A: 445] and those with a prothrombotic tendency (thrombophilia, family history of VTE, other) [Group-C: 165] there were no significant differences in general; those undergoing IVF [Group-B: 132] were of a higher age (37.2 ± 5.1), one out of five had multiple pregnancy (30, 22.7%) and concomitantly received aspirin more frequently (30.3%). In terms of outcomes, again there were differences between the groups; specifically, in Group B the frequency of GVCs was almost twice as high in comparison with that of Group A and Group C, mainly driven by differences in IUGR and early pregnancy loss events. The characteristics of the study population are presented in Table 1.

**Table 1:** Characteristics of the study population organized according to the groups and according to demographic/medical record, anticoagulation treatment, and outcomes (gestation related and coagulation related)

		**Group A *N* = 445**	**Group B *N* = 132**	**Group C *N* = 165**	**p**
Baseline	Age (mean ± SD)	33.5 ± 4.6	37.2 ± 5.1	32.5 ± 4.4	** < .0001**
	BMI (mean, SD)	24.4 ± 3.9	24.7 ± 3.8	24.4 ± 4.3	0.4281
	No. of foetuses at the observed gestation (N, %)				** < .0001**
	1	434, 97.5%	102, 77.3%	158, 95.8%	
	≥ 2	11, 2.5%	30, 22.7%	7, 4.2%	
	High risk Thrombophilia (positive cases)	10.10%	9.90%	10.30%	0.9916
Treatment	Mean Duration of LMWH (months)	8.7 ± 1.3	8.7 ± 1.7	8.3 ± 1.6	** < .0001**
	Fixed Prophylactic Dose	58.90%	50%	52.10%	0.1100
	Weight Adjusted prophylactic dose	32.40%	38.60%	37.00%	0.3092
	Therapeutic dose of LMWH	8.80%	11.40%	10.90%	0.5663
	Concomitant Use of ASA	18.20%	30.30%	12.10%	**0.0003**
Outcomes	Caesarian	79.70%	91.70%	65.50%	** < .0001**
	Live Birth	99.10%	97.00%	99.40%	0.1039
	Gestational Vascular Complications (Total) (N, %)	37 (8.3%)	23 (17.4%)	16 (9.7%)	**0.0097**
	IUGR	13 (2.9%)	8 (6.1%)	5 (3.0%)	0.2114
	Preterm Labor	19 (4.3%)	12 (9.1%)	10 (6.1%)	0.0978
	Fetal Death	1 (0.2%)	1 (0.8%)		0.5981
	Early pregnancy loss/abortion	3 (0.7%)	2 (1.5%)		0.5427
	Pre-eclampsia/eclampsia	1 (0.2%)		1 (0.6%)	0.6301
	VTE [VTE postpartum]^a^	3 (0.7%) [3 (0.7%)] (5 women)	0 [1 (0.8%)] (1 woman)	1 (0.6%) [5 (3.0%)] (6 women)	0.0632
	Bleeding	6 (1.3%)	5 (3.8%)	1 (0.6%)	0.0728

^a^ Indicates the VTE events during gestation, the figures within square brackets depict the VTE events during the postpartum period, while the figures in parentheses indicate the number of women involved

### Variation of the risk among the studied groups

The mean score antepartum was higher for women in Group B (3.3 in comparison with 1.9 and 2.0 in Group A and Group C respectively). Moreover, the risk score increased significantly postpartum for all Groups. The descriptive characteristics of the VTE risk score for each group, are presented in Table 2.

**Table 2:** Descriptive characteristics of score points for each group. SD: Standard Deviation, Min: minimum, Max: Maximum, Q1: Quartile 1, Q3: Quartile 3

		**Risk antepartum**					**Risk postpartum**					
Group	N	**Mean**	**SD**	**Median**	**Q1**	**Q3**	**Mean**	**SD**	**Median**	**Q1**	**Q3**	**p**
Group A	445	1.9	2.2	1	1	2	3.1	2.3	2	2	4	< 0.0001
Group B	132	3.3	2.1	3	2	4	4.6	2.3	4	3	6	< 0.0001
Group C	165	2.0	1.7	2	1	3	3.2	1.9	3	2	4	< 0.0001

Afterwards, the differences in the risk scores between the three groups were compared by two methods: a) as an arithmetic score and b) as a qualitative measure (i.e. after being characterized as: low, intermediate and high).

**Fig. 1:**
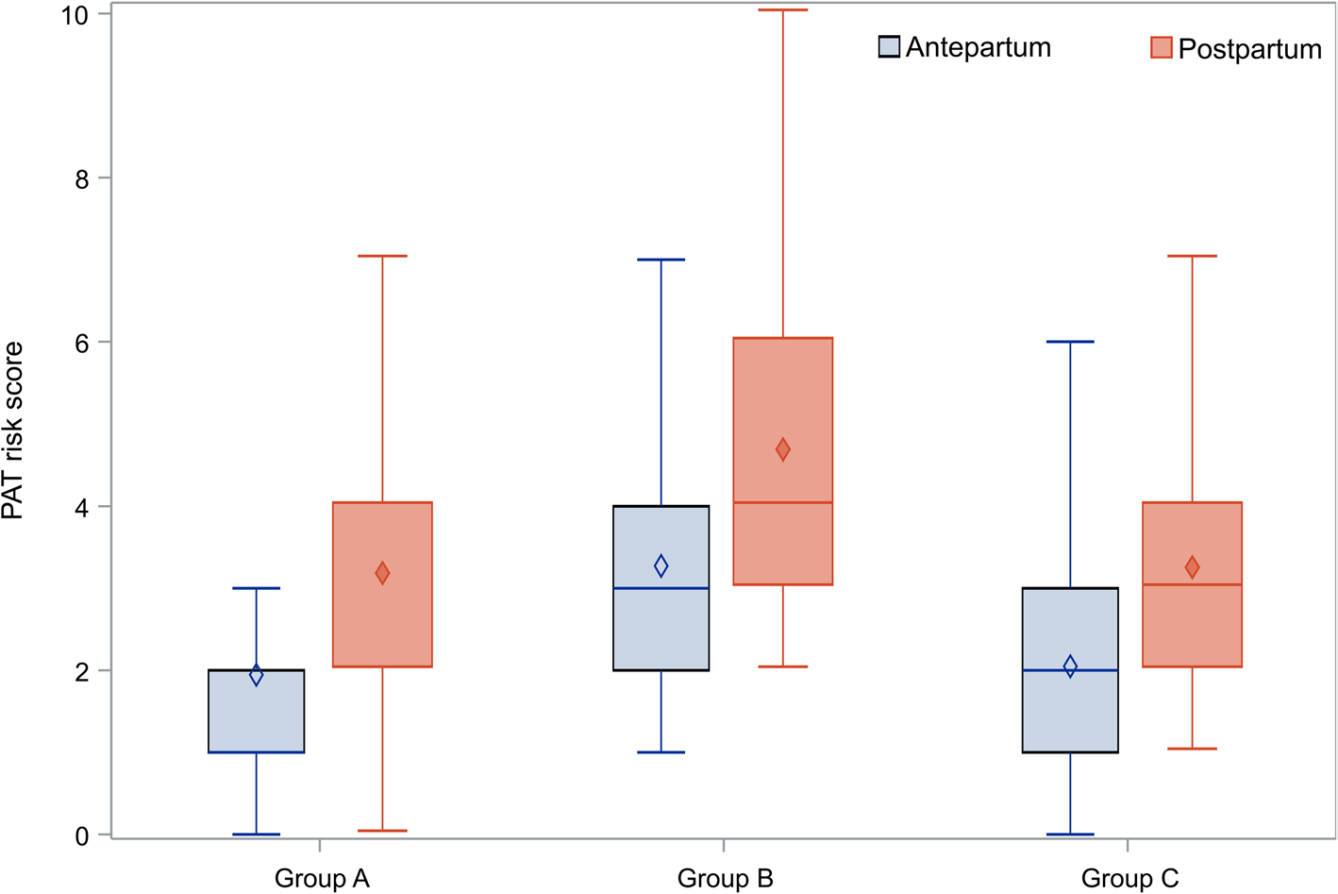
Box and whisker plots of the total risk for the groups studied. For each group the lower part of the box indicates the 1^st^ quartile, while the upper part represents the 3^rd^ quartile; the lines within the boxes are for the median values and the diamond symbols are for the mean values; the horizontal lines at the lower and upper part of the whiskers indicate the minimum and maximum observations after excluding outliers

A graphical representation of the results of the first approach (arithmetic score) is presented in Fig. 1. The statistical test indicated a significant difference in the scores between the three groups both before (*p* < 0.0001) and after birth (*p* < 0.0001).

The results of the investigation by score characterization that we conducted, are presented in Table 3. The data for risk antepartum and risk postpartum are presented separately. It is interesting to note that group B had a higher percentage of women at high risk (25.76% and 60.61% before and after delivery) compared to group A (18.88% and 28.31%) and group C (15.15% and 28.48%). A significant difference (*p* < 0.0001) was found in all comparisons between group B vs. group A or C.

**Table 3:** Cross tabulation of the risk characterization for each individual group. Each cell depicts the number of cases and the relevant percentage

	**Risk antepartum**			**Risk postpartum**				
	**High**	**Intermediate**	**Low**	**High**	**Intermediate**	**Low**	**Total**	**p**
**Group A**	84 (18.9%)	23 (5.2%)	338 (76.0%)	126 (28.3%)	61 (13.7%)	258 (58.0%)	445	< 0.0001
**Group B**	34 (25.8%)	39 (29.6%)	59 (44.7%)	80 (60.6%)	33 (25.0%)	19 (14.4%)	132	< 0.0001
**Group C**	25 (15.2%)	18 (10.9%)	122 (73.9%)	47 (28.5%)	46 (27.9%)	72 (43.6%)	165	< 0.0001
**Total**	143	80	519	253	140	349	742	< 0.0001

Actually, group A and C have a lower distribution of the women in the high or intermediate risk group, while in group B 55.3% of the women were at intermediate or high risk before delivery and this percentage increased further after delivery (85.6%). The chi-square test also proved that there is a higher percentage of women at high or intermediate risk in group B compared to C before birth (55.3% vs.26.1% respectively, *p* < 0.0001, OR: 3.5, 95% CI: 2.2 – 5.7) and similarly after birth (85.6% vs. 56.4%, OR: 4.6, 95%CI: 2.6–8.2, *p* < 0.0001).

When comparing group B with the combined figures for groups A and C, we found that before birth the odds for Intermediate or high risk in group B were 3.8 times higher (95% CI: 2.6 – 5.6, *p* < 0.0001) than the odds in groups A and C combined (see Table 4), while after birth the OR was almost twice as high (7.0, 95% CI: 4.2–11.7, *p* < 0.0001).

**Table 4:** Cross tabulation of the risk, intermediate and high vs. low for the groups A&C combined vs. group B. Each cell depicts the number of cases and the relevant percentage

	**Risk antepartum**		**Risk postpartum**			
	**Intermediate or High**	**Low**	**Intermediate or High**	**Low**	**Total**	**p**
**Group A or C**	150 (24.6%)	460 (75.4%)	280 (45.9%)	330 (54.1%)	610	< 0.0001
**Group B**	73 (55.3%)	59 (44.7%)	113 (85.6%)	19 (14.4%)	132	< 0.0001
**Total**	223	519	393	349	742	< 0.0001

### Coexistence of risk factors in the groups studied

**Fig. 2:**
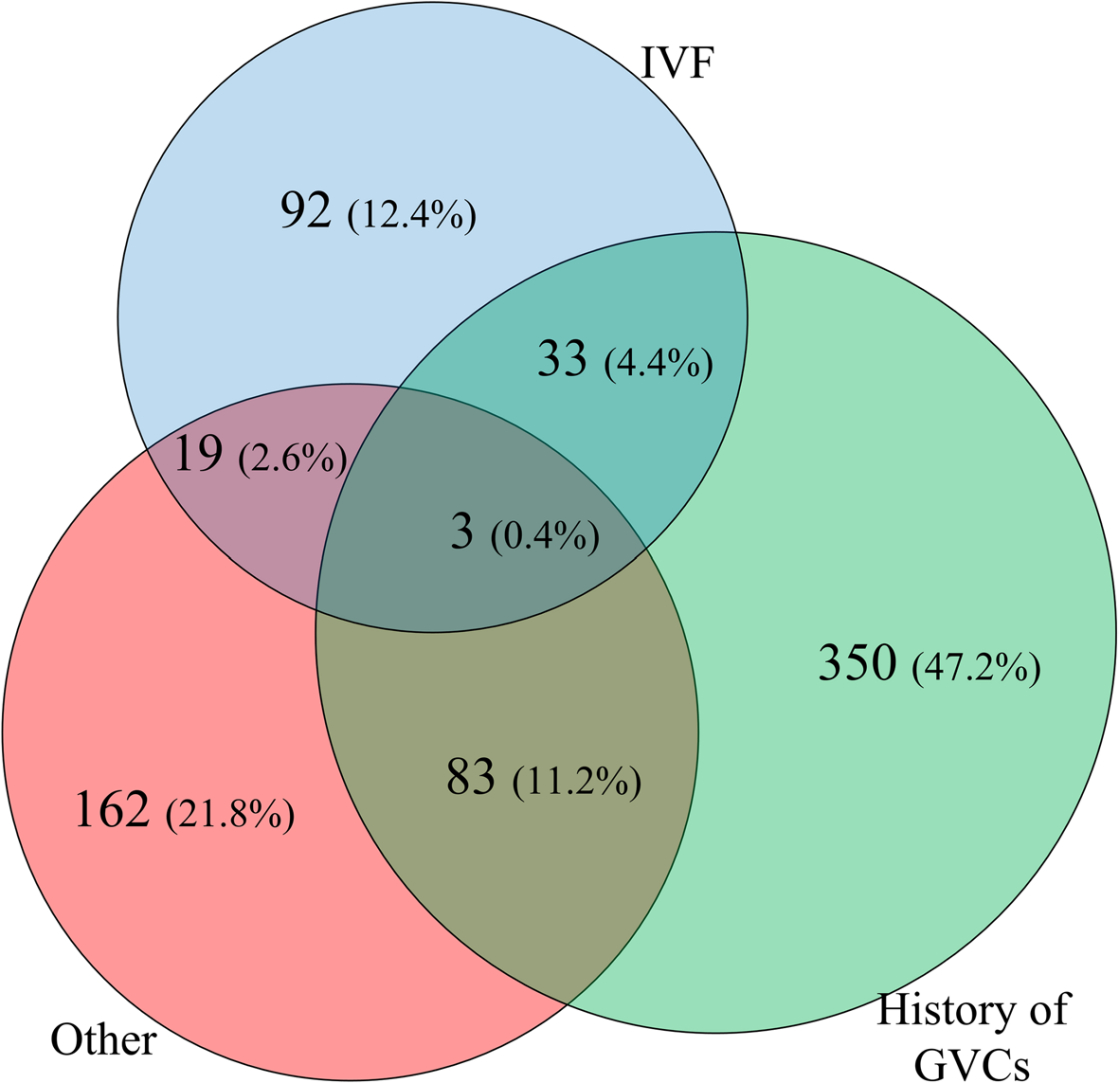
Venn diagram depicting the distribution of women into the various categories according to their reason for inclusion

In actual fact, the separation of women into the three groups studied is not the only one possible since women may have more than one reasons for being included in the study, and thus they could have potentially been included in more than one group. Within the source study [17], women were assigned to a group on the basis of one basic reason only. However multiple reasons could exist and the distribution of the women according to these reasons is depicted in Table 5. Actually 81.4% of the women in the study had a single reason while the remaining 18.6% had multiple reasons and thus these women had an increased thrombotic load. A graphical diagram depicting the overlapping of inclusion reasons in this study is depicted in Fig. 2.

**Table 5:** Reasons harbored by individual women

Reason	N	%
IVF only	92	12.4%
History of GVCs ^a^ only	350	47.2%
Other only ^b^	162	21.8%
IVF & History of GVCs	33	4.4%
IVF & other	19	2.6%
History of GVCs & other	83	11.2%
IVF & History of GVCs & other	3	0.4%

^a^ Early pregnancy loss, fetal death, eclampsia/preeclampsia and IUGR

^b^ Including family history of thrombophilia and/or thrombophilia

### Observed risk components

With regard to the two major risk components that go into the calculation of the PATrisks score, that is a) pre-existing risk factors and b) obstetric risk factors, the distribution of the population studied according to the individual components is depicted in Table 6. A small percentage 11% had no risk factor, while 38% and 8% had only obstetric and pre-existing risk factors respectively; moreover, almost 43% of the women in the study, had simultaneously more than a single factor, that is, they had both pre-existing and obstetrics risk factors.

**Table 6:** Risk component harbored by the participating women

**Risk component**	N	%
none	83	11.19%
Pre-existing	61	8.22%
Obstetrics	280	37.74%
Pre-existing & obstetrics	318	42.86%

### Risk score and thrombotic events

**Fig. 3:**
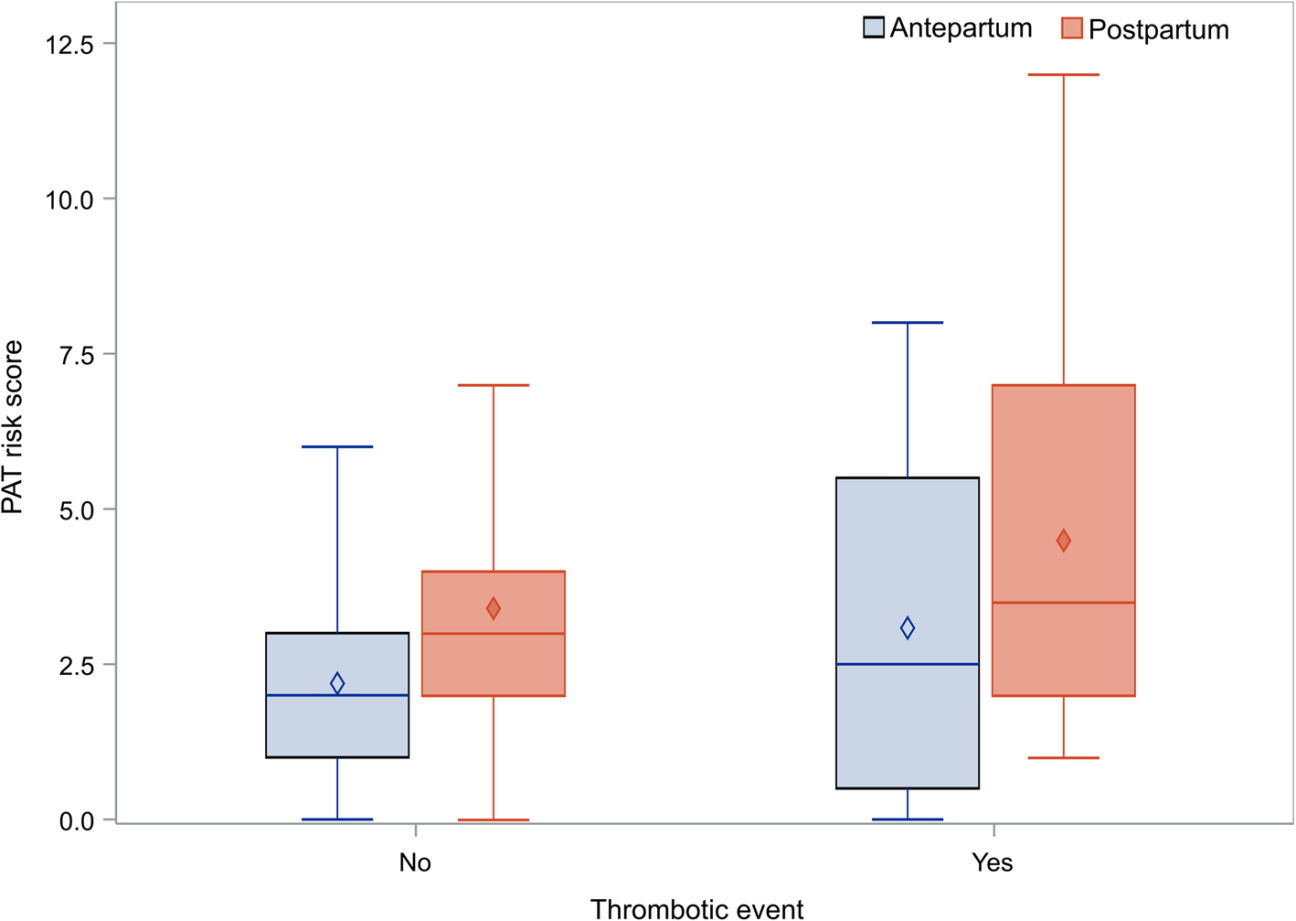
Box and whisker plots of the risk score for the women who did and did not experience thrombotic event during the study. Box limits show the 1^st^ and 3^rd^ quartiles, whisker limits show the minimum and maximum values (excluding outliers), while the lines and diamonds within the boxes correspond to the median values and the mean values respectively

In total 12 (1.6%) out of 742 women included in our analysis experienced thrombotic events, whether pre- or post-partum, during the study period (see Table 1 [one woman had two thrombotic events]). The median value of the risk score for the women experiencing thrombosis was 2.5 (Q1-Q3: 0.5–2.5) before birth and this score rose to a median value of 3.5 (Q1-Q3: 2–3.5) after delivery. There was no significant difference in the score between the women who did and did not experience such events whether before (*p* = 0.4319) or after delivery (*p* = 0.4004) (see Fig. 3 for the relevant diagram), probably due to the anticoagulation prophylaxis given to all participating women. It is interesting to note that some women with high risk (score > 10) did not experience thrombotic events. Among women who did experience thrombosis, five had a low score, one had an intermediate score (i.e. 3) and the remaining 6 had a score higher than 4 (high risk).

The analysis of the six women with a low or intermediate score showed that two were in group A and four in group B while none were in group C. Out of these six women, one had a family history for thrombosis, one had a multiple pregnancy, one received blood transfusion and four had low risk thrombophilia; in addition, the one with the family history of VTE had a BMI of 42.1. Five out of the six thrombotic events were postpartum when no prophylaxis was applied, precisely because they had low risk score for VTE.

### Analysis of thrombophilia factors observed in the various groups

Of special interest is the distribution of high-risk thrombophilia observed in the study.

The figures indicating the presence of thrombophilia risk factors per Group and in Total are given in Table 7. Women with APLA who were in group B had a higher PATrisks score.

**Table 7:** Thrombophilia risk factors grouped into low- and high-risk types for the three study groups. Bold *p*-values indicate statistical significance

		Number of cases, median [q1—q3]			Total, *p*-value
	Factor	Group A	Group B	Group C	
High risk inherited	Reduced levels of ΑΤ (< 70%)	6, 8.5 [6—8.5]	2, 7.5 [7—7.5]	3, 7 [4 - 7]	11, 0.7659
	Reduced levels of PC(< 70%)	7, 7 [6 - 7]	1, 11 [11 - 11]	1, 11 [11 - 11]	9, 0.116
	Reduced levels of PS_F (< 60%)	11, 7 [6 - 7]	4, 7.5 [6.5—7.5]	3, 7 [6 - 7]	18, 0.6525
	Reduced levels of PS_C (< 60%)	9, 6 [6 - 6]	2, 10 [8 - 10]	3, 4 [4 - 4]	14, 0.1403
	FV Leiden homozygous aa	7, 6 [4 - 6]	2, 7 [5 - 7]	7, 7 [6 - 7]	16, 0.6533
	FI20210 homozygous aa	2, 7 [6 - 7]	1, 8 [8 - 8]	1, 5 [5 - 5]	4, 0.3247
	FV Leiden and FII20210 combined heterozygous ga	11, 7 [7 - 7]	1, 12 [12 - 12]	0, [ -]	12, 0.0973
High risk acquired	APLA syndrome	56, 6 [6 - 6]	16, 8 [8 - 8]	11, 6 [4 - 6]	83, < **0.0001**
Low risk inherited	FV Leiden heterozygous ga	136, 4 [4 - 4]	32, 6 [5.5—6]	59, 4 [2 - 4]	227, < **0.0001**
	FI20210 heterozygous ga	67, 4 [4 - 4]	21, 6 [5 - 6]	30, 4 [2 - 4]	118, **0.0038**
Other low risk	MTHFR	297, 4 [3 - 4]	95, 6 [5 - 6]	114, 4 [2 - 4]	506, < **.0001**
	PAI4G	148, 4 [3 - 4]	44, 6 [5 - 6]	80, 4 [2 - 4]	272, < **.0001**
	Factor XII above normal range (50–150)	6, 3 [1 - 3]	4, 11 [10 - 11]	3, 3 [3 - 3]	13, **0.0167**
	Factor VIII above normal range (50–150)	8, 7 [3.5—7]	6, 11 [10 - 11]	3, 8 [4 - 8]	17, **0.0454**
	Other low-ris	51, 3 [1 - 3]	24, 5.5 [5—5.5]	33, 3 [1 - 3]	108, < **.0001**

Out of the total study population of 742 women, 149 of them (20.1%) had high-risk thrombophilia (note that many women had multiple thrombophilias); specifically, there were 96 of them in group A, 26 in group B and 27 in group C, representing 21.6%, 19.7% and 16.4% out of each individual group population respectively. No statistically significant difference was found in the percentages of population with high-risk thrombophilia in the groups studied (*p* > 0.05 in all comparisons). Low-risk thrombophilia was observed in 83.7% of the total population, that is 82.3% in group A, 81.1% in group B and 89.7% in Group C; no significant difference was found between groups A and B (*p* = 0.7554); however, there was noticeable differences between groups A and C (OR: 1.9, 95% CI: 1.1—3.3, *p* = 0.0248) as well as between groups B and C (OR: 2.0, 95% CI: 1.0—4.0, *p* = 0.0338). However, the low risk factors with well-established association with VTE such us the heterozygous mutations for FV Leiden and FII 20,210 were found in 39.1% and 21.1% of the total population respectively (difference 18%, 95%CI: 13.3–22.6%, *p* < 0.0001). Finally, none of the thrombophilia risk factors presented in Table 7 was observed in 102 of the women (13.7% of the total population); these women were distributed as follows: 66 in group A (14.8% of the group population), 23 in group B (17.4%) and 13 in group C (7.9%).

## Discussion

### Summary of the key findings

In this post-hoc analysis, we retrospectively evaluated thrombotic risk in women who received LMWH based on clinical experience hoping to optimize their pregnancy outcome, so that we could investigate whether that practice was justified according to the PATrisks scoring system. The arithmetic mean score antepartum was higher for women in Group B (3.3) in comparison with that in Group A and Group C (1.9 and 2.0 respectively). This risk score increased significantly postpartum for all Groups. On the basis of the score characterization as high, intermediate and low, group B had a statistically significant higher percentage of women at high risk (25.76% and 60.61%, before and after delivery) compared to group A (18.88% and 28.31%) and group C (15.15% and 28.48%) in all comparisons. In relation to the major risk components that go into the calculation of the PATrisks score, a small percentage of women (11%) had no risk factor, while 38% and 8% had only obstetric and pre-existing risk factors respectively; moreover, almost 43% of women had more than a single risk factor type simultaneously. In total, 12 (1.6%) out of the 742 women included in our analysis experienced thrombotic events during the study. The median value of the risk score for the women experiencing thrombosis was 2.5 (Q1-Q3: 0.5–2.5) before birth and this score increased to a median value of 3.5 (Q1-Q3: 2–3.5) after delivery. No significant difference was found in the score between the women who did and did not experience such events either before (*p* = 0.4319) or after delivery (*p* = 0.4004).

Out of the total study population, 149 (20.1%) women had high risk thrombophilia; specifically 21.6% in group A, 19.7% in group B and 16.4% in group C. Absence of thrombophilia risk factors was observed in 102 women (13.7% of the total population), specifically in 14.8%, 17.4% and 7.9% of the women in groups A, B and C respectively.

Although an established VTE in pregnancy may be successfully treated with therapeutic doses of heparin, prevention is preferable to cure because of the high mortality and long-term morbidity associated with established disease [21, 22]. LMWHs can be used safely in pregnancy and in breastfeeding with no adverse effects on the fetus or the neonate. Medical prophylaxis against thrombosis starts during pregnancy and is generally continued for about six weeks following delivery due to the risk of thrombosis which peaks during the postpartum period. Because of their efficacy and good safety profile, the use of low molecular weight heparins for thromboprophylaxis in pregnancy has increased greatly in recent years. Their use has also increased in women at high risk of GVCs and for the optimization of ART outcomes in anticipation of the scientific evidence in favor of their use becoming available. Clinicians have proposed these treatments on the grounds of biological plausibility and on the basis of extrapolation from antiphospholipid syndrome [23]. There is also increasing evidence for the use of heparin in women with pregnancy complications mediated by the placenta (as can be seen from successful previous pregnancy outcomes), while this is not the case for women with thrombophilic defect alone [24].

In our cohort, administration of LMWH for VTE prevention was found to be both efficacious and safe; only 1.6% out of the total women population included in our analysis experienced thrombotic events (whether pre- or post-partum) during the study, and the prevalence of bleeding events was also low.

From our analysis we found that despite the fact that the administration of LMWHs in all groups was based on clinical experience, in one out of four women for Groups A & C and in more than half of women in Group B this was justified antepartum by the scoring tool since they were found to have intermediate or high risk for VTE. Additionally, the administration of LMWHs postpartum was justified to a higher extend; specifically, almost half of the women in Groups A & C and more than eight out of ten in Group B were found to have intermediate or high risk, after birth. It is interesting to note that, regardless of which group they had been allocated to, the number of women with intermediate or high score was almost twice as high postpartum (393) as antepartum (223).

With regard to the major risk components that go into the calculation of the PATrisks score only one out of ten women had no risk factors at all, while 38% and 8% of them had only obstetric or pre-existing risk factors respectively; moreover, almost 43% of the women, had both pre-existing and obstetrics risk factors simultaneously. Those findings also provide, at least partially, justification for the administration of LMWH in the population under study.

Robertson et al. [25] conducted a large systematic review to assess the overall relationship between all major thrombophilia and adverse pregnancy outcomes, which comprised 79 studies, primarily consisting of case–control studies. All inherited thrombophilia defects were associated with venous thromboembolic disease (VTE), homozygous/ heterozygous FV G1691A and FII G20210A were associated with pregnancy loss < 24 weeks [Odds ratio (OR) 2.71, 1.68, 2.49 respectively], heterozygous FV G1691A, FII G20210A, and PS with pregnancy loss > 24 weeks (OR 2.06, 2.66, 20.09), heterozygous FV G1691A and FII G20210A with PE (OR 2.19, 2.54), and heterozygous FV G1691A and FII G20210A with PA-VTE (OR: 4.70, 7.71).

That being so, in the PATrisks tool all the “high risk thrombophilias” (Table 7) are rated with 3 points, and from the “low risk thrombophilias” only the heterozygous mutations for FV Leiden and FII 20,210 are rated with 1 point. The “other risk thrombophilias” are not scored in the PATrisks tool because their association with the occurrence of VTE is not well established. Out of all the women in the study, one out of five had high risk thrombophilia and one out of ten had APLA; both conditions during pregnancy also provide a rationale for the use of thromboprophylaxis. Additionally, the low risk factors such us the heterozygous mutations for FV Leiden and FII 20,210 which were found in 39,1% and 21,1% of the total population respectively, again provides some rationale for primary prophylaxis from thrombotic events in the context of pregnancy and puerperium only if there are other preexisting or/and obstetrics risk factors.

In terms of pregnancy outcomes and despite the quite high prevalence of various thrombophilic disorders with known association with pregnancy complications, the frequency of GVCs in the pregnancies examined was low overall; the most frequently observed GVCs were preterm labor and IUGR.

In our days, assisted reproductive technologies (ART) are widely used in couples with fertility problems and low-molecular-weight heparin (LMWH) is routinely administered in various cases to improve ART outcomes. The live birth rate in Group B of our cohort was 97% which is quite high for women undergoing assisted reproduction techniques, indicating a possible favorable effect of LMWH administration in successful ART outcomes. Live birth rates were also high (higher than 99%) in both Groups A & C.

This post-hoc analysis also shares the limitations, as well as the advantages, of most observational studies. By its design, the study involves a broad range of everyday/routine clinical approaches, and there is limited specific focus for the inclusion in Groups of the participating women. Thus, biases of an unknown nature may have colored the results. This study does not feature the typical separation of patients into case and control groups—instead, the analysis involved all patients in an intention-to-treat manner. This list of limitations is by no means exhaustive. However, in the authors’ opinion, this study has the potential to capture the “in vivo” conditions of a common clinical obstetrics setting. We recognize that a control group of patients would strengthen the analysis. As we mentioned before, the study is retrospective, and it is a post hoc analysis of our previous work [17]. Pregnant women had received LMWH hoping to achieve a good pregnancy outcome. Their data were analyzed retrospectively to determine whether LMWH could provide to some of them an additional benefit as thromboprophylaxis, according to the PATRisks score which is based on RCOG guidelines. These guidelines have emerged from older, large studies which recognize the increased risk of VTE in pregnancy and postpartum [1–3, 18]. In the future, we would like to study the prospective evaluation of the PATrisks score in pregnancy.

## Conclusions

LMWHs are widely prescribed during pregnancy for a variety of indications with no proven scientific basis for all of them, due to the lack of randomized trials. However, a considerable percentage of women with pregnancy complications, those undergoing IVF, and those carrying prothrombotic tendencies who are treated with LMWH were already at VTE-risk according to PATrisks and might have derived an additional benefit from LMWH in the form of VTE prevention. The rational use of these drugs should be optimized by establishing and implementing routine risk assessment for all pregnant women and by providing the necessary education to healthcare professionals.

## Data Availability

The datasets used and/or analyzed during the current study are available from the corresponding author on reasonable request.

## Abbreviations

ACL: Anticardiolipin antibodies
APLA: Antiphospholipid antibodies
ART: Assisted Reproduction Techniques
ASA: Acetylsalicylic acid
AT: Antithrombin
B2GP: B-2 glycoprotein antibodies
BMI: Body mass index
DVT: Deep vein thrombosis
CS: Caesarian section
FGR: Fetal growth restriction
GVCs: Gestational vascular complications
IUGR: Intrauterine growth retardation
IVF: In Vitro Fertilization
LAC: Lupus anticoagulant
LMWHs: Low molecular weight heparins
OR: Odds Ratio
PA-VTE: Pregnancy-associated Venous thromboembolism
PC: Protein C
PE: Pulmonary embolism
PS: Protein S
Q1: Quartile 1
Q2: Quartile 2
RCOG: Royal College of Obstetricians and Gynecologists
SD: Standard Deviation
VTE: Venous thromboembolism
